# Case of Acute Traumatic Tetanus; with Some Observations, as Given in a Clinical Lecture

**Published:** 1827-04

**Authors:** H. Earle

**Affiliations:** St. Bartholomew's Hospital.


### TRAUMATIC TETANUS.

*Case of Acute Traumatic Tetanus; with some Observations, as given in a Clinical Lecture, by* H. Earle, Esq. F.R.S. &c. (St. Bartholomew's Hospital.)

Stephen Thomas Mitchell, ætatis twenty, a glass-cutter, who had enjoyed good health all his life, was admitted into Pitcairn's Ward, on Monday, February 26th, at eight P.M. Six days before, while wrestling, he wounded the sole of his foot with a blunt rusty nail. He applied a linseed poultice to the part, and took some aperient medicine. On Sunday the 25th, after taking a hearty meal, he perceived a slight pain and stiffness in the back, and in a short time the muscles of the neck became rigid. When admitted into the hospital, the muscles of the jaw and abdomen were also affected. His bowels had not been opened for thirty-six hours; pulse eighty-two, full and hard. The wound in the foot had healed, and there was no surrounding inflammation or tenderness. The lower extremities were free from spasm. At half-past eight, Calomel gr. v. Jalap gr. xv. were with difficulty swallowed. At twelve, the same dose was repeated; and in about two hours after he took Infusi Sennæ Comp. ℥ij. During the night he passed five dark offensive evacuations, without experiencing any mitigation.

On the 27th, at nine A.M. he was decidedly worse; the jaw more closed, and the spasmodic affection constant, with more frequent exacerbations. He complained of great pain in the abdomen and at the back of his neck, and the muscles of the lower extremities were slightly affected. He was ordered Calomel gr. j. Jalapæ gr. v. every four hours. At half-past twelve, as no improvement had taken place, he was directed to take m. v. of Hydrocyanic Acid, and gradually to increase the dose until a decided effect was produced. At half-past two, twenty ounces of blood were taken from his arm, with temporary relief; his pulse varying from 120 to 150. At three o'clock, ten ounces more blood were taken, and he appeared easier for a short time. He had expressed a strong desire to be bled. At half-past three, the spasms were as violent as before: m. viij. of Hydrocyanic Acid were given, and ten more at four, without producing any sensible effect. At five P.M. the dose was increased to m. xx. and he became easier for an hour and a half: the spasms were less violent, and not so frequent; and he slept for a short time. At half-past six, he again complained much of his neck and abdomen; his pulse was softer, and about 130. He had continued to take the calomel and jalap, but had passed no evacuations since the morning. He attempted to take a dose of house physic, but could not swallow it. At seven, he took m. xx. more of the Hydrocyanic Acid, but without experiencing even temporary relief. At nine, the spasms were more violent, and his pulse was too rapid to be counted. He had perspired profusely the whole day; but, as no water had passed, a catheter was introduced, and about six ounces of high-coloured urine were drawn off. He gradually got worse, the muscles of the throat became violently affected, and he died in convulsions about midnight.

*Post-mortem examination.*—The pia mater was, perhaps, a little more vascular than natural; there were more red spots in the medullary substance of the brain than are met with in its healthy state; the spinal cord was healthy. There was extensive inflammation of the left pleura; the left lung was gorged with blood, and much inflamed. The sympathetic nerve, where it was in contact with the pleura, was very vascular. The right pleura, lung, and sympathetic nerve, were not so much inflamed as the left. There were several spots of effused blood between the pleura and diaphragm on the left side; also between the pleura and the aorta. The aorta was filled with fluid blood, its external coat was perfectly healthy. There was about an ounce of fluid in the pericardium. That part of the pericardium which is reflected over the right auricle was somewhat inflamed, and under it were several spots of effused blood; the right auricle and ventricle were filled with coagulated blood.

In the abdomen were observed recent adhesions between the stomach and liver. The peritoneal covering of both organs was much inflamed; as was the liver itself, and the gall-ducts in its substance were filled with bile. The spleen was tuberculated and inflamed. The mesenteric glands were numerous and enlarged. The mesentery was inflamed. The mesorectum had several spots of effused blood between its layers, and was inflamed. The alimentary canal was healthy; the stomach and small intestines contained mucus; the large intestines were filled with a very offensive dark-coloured feculent matter. All the nerves in the abdomen looked healthy.

At the bottom of the wound in the foot was a small piece of skin, about the eighth of an inch in diameter, which had been apparently pushed in by the nail. The internal plantar nerve, before it had divided into the two branches which supply the great toe and the toe next it, was completely torn through, and each extremity of the nerve was bulbous and vascular: every other part of the nerve appeared perfectly healthy. The theca binding down the tendons of the great toe was wounded.

This case affords another melancholy proof of the rapidly destructive effect of this uncontrollable disease. In the present instance it attacked suddenly, and speedily attained its most violent degree, affording no time for the administration of a milder plan of treatment capable of influencing the state of the secretions, which were obviously much deranged. It may be urged, that the removal of the injured part might in this instance have been beneficial, as the wounded nerve was bulbous and inflamed. To this it may be answered, that there was no symptom during life indicating any such affection, and the instances of ineffectual operations abound too much in the annals of military surgery to warrant a repetition. Even Baron LARREY, who advocates this practice, expressly confines it to mere chronic affections; in which cases sufficient time is generally allowed to employ other and less severe measures.

I was induced to employ the hydrocyanic acid from its powerful influence on the nervous system. The doses in which it was given, without producing any specific effect, prove how insensible the nervous system was to the operation of medicine. I was further induced to make trial of this remedy, from the numerous list of fatal cases which exist in the records of surgery, where opium and other more common medicines had been most extensively employed. As it was not given in sufficient doses sensibly to affect the nervous system, it can hardly be said to have failed, and, in the absence of better remedies, it may yet deserve a further trial.

In such acute affections, which in rapidity and severity very nearly resemble hydrophobia, it is justifiable to employ any new plan of treatment which affords the slightest ground for hope. I would venture to suggest in such cases the employment of Strychnine, in doses to affect the nervous system; and further, I should like some experiments to be tried on animals affected with tetanus, of the effect of carbonic acid gas, administered to an extent to produce temporary suspension of animation, which might be restored by artificial respiration.

I merely throw out these remarks as hints which may be worth entertaining, as the common beaten track has so often led to fatal terminations.

The dissection in this case was interesting, as it rarely happens that there are such unequivocal marks of inflammation. The blood which was taken coagulated firmly, but exhibited no appearance of inflammation. It has been stated by CULLEN, and even by recent authors, that the blood in tetanus will not coagulate, but appears broken down. I have met with cases in which the blood has exhibited the strongest marks of inflammation. In one case, which I published in the Medico-Chirurgical Transactions, where above one hundred ounces were taken, the blood was buffed and cupped to the last.

*George-street, Hanover-square; March,* 1827.

